# A Systematic Review of the Antecedents and Prevalence of Suicide, Self-Harm and Suicide Ideation in Australian Aboriginal and Torres Strait Islander Youth

**DOI:** 10.3390/ijerph16173154

**Published:** 2019-08-29

**Authors:** Joanne M. Dickson, Kate Cruise, Clare A. McCall, Peter J. Taylor

**Affiliations:** 1 School of Arts and Humanities, Division of Psychology, Edith Cowan University, Joondalup 6027, Australia; 2 Division of Psychology & Mental Health, University of Manchester, Manchester Academic Health Sciences Centre, Manchester M13 9PL, UK

**Keywords:** Aboriginal and Torres Strait Islander youth, suicide, self-harm, suicide ideation, systematic review

## Abstract

Suicide and self-harm represent serious global health problems and appear to be especially elevated amongst indigenous minority groups, and particularly amongst young people (aged 24 years or younger). This systematic review investigates for the first time the antecedents and prevalence of suicide, self-harm and suicide ideation among Australian Aboriginal and Torres Strait Islander youth. Web of Science, PubMed, PsychINFO, CINAHL databases and grey literature were searched from earliest records to April 2019 for eligible articles. Twenty-two empirical articles met the inclusion criteria. The data confirmed that indigenous youth in Australia have elevated rates of suicide, self-harm and suicidal ideation relative to the nonindigenous population. Risk factors included being incarcerated, substance use and greater social and emotional distress. Notably, though, information on predictors of suicide and self-harm remains scarce. The findings support and justify the increasing implementation of public health programs specifically aimed at tackling this crisis. Based on the review findings, we argued that Aboriginal communities are best positioned to identify and understand the antecedents of youth self-harm, suicide ideation and suicide, and to take the lead in the development of more effective mental health preventive strategies and public policies within their communities.

## 1. Introduction

Suicide is the cause of over 800,000 deaths annually around the globe [1]. The World Health Organization (WHO) has urged that suicide prevention be given a higher priority on the global public health agenda [1]. Suicide attempts and other acts of self-harm are even more prevalent and are associated with an elevated risk of eventual death by suicide, as well as reduced lifespan more generally [2,3,4]. Here we use the term self-harm to encompass both suicide attempts and non-suicidal self-injury ((NSSI) e.g., self-cutting as a means to manage or cope with difficult emotional states) [5,6,7,8]. Self-harm and suicidal ideation are markers of considerable emotional distress, often associated with other psychological difficulties, and so remain important clinical outcomes in their own right [9,10,11,12]. It has been recognised that suicide rates are often elevated amongst Indigenous populations, including Australian Aboriginal and Torres Strait Islander peoples [1,13]. Young people can be especially vulnerable to some of the factors that contribute to risk of suicide and self-harm. Data on the prevalence of suicide, self-harm, and suicidal ideation in these populations is important in determining the level of clinical need and to better understand the challenges faced. However, information on the specific risk factors and predictors of suicide and self-harm risk among young Australian Aboriginal and Torres Strait Islanders is also vital for establishing and supporting prevention and intervention strategies. Knowledge of what psychological, social, intergenerational or contextual factors contribute to elevated risk may, for example, identify targets for specific prevention and therapeutic interventions and public health agendas and campaigns. The current systematic review therefore investigates the prevalence, correlates and predictors of suicide, self-harm and suicidal ideation risk amongst Aboriginal and Torres Strait Islander young people in Australia.

Indigenous peoples generally face several stresses and challenges that may account for heightened suicide and self-harm rates, including challenges around acculturation and minority group status, discrimination, socioeconomic deprivation, poverty, unemployment and inequalities in health systems [1,14,15]. Mental health problems such as depression and substance use have been noted as prevalent amongst Aboriginal and Torres Strait Islander peoples and may further account for the risk of suicide and self-harm [16,17,18]. Self-harm is known to peak during adolescence [19]. Where indigenous communities are facing these stressors, young people may be especially at risk, particularly where a lack of support is available in the community [20]. Further, given the high incidence of suicide among some Indigenous communities, many Aboriginal young people have been witness to the death of family members and friends [21]. Potentially high levels of exposure to death and suicide among indigenous communities has been argued to result in a ‘normalisation’ of self-harm and suicide [20]. In conjunction with a lack of support available to Aboriginal youth, the ‘normalisation’ of suicide may fuel a young person’s perception that suicide is a possible solution to challenging life circumstances [21].

This systematic review aims to examine the prevalence and antecedents of suicide, self-harm and suicide ideation among Australian Aboriginal and Torres Strait Islander youth. The review’s focus on young Aboriginal and Torres Strait Islanders under the age of 25 years is in line with the World Health Organization’s (WHO) definition of ‘youth’ as those aged between 15 to 24 years and adolescence between 10 to 19 years [22]. The review includes Aboriginal and Torres Strait Islander children in light of increasing rates of suicide among this age group [13]. It is informative to study suicide prevalence data in order to understand the breadth of this crisis among Aboriginal and Torres Strait Islander youth and to inform ongoing mental health agendas. Suicide and self-harm in this population arguably extends beyond mental health, reflecting broader social, cultural and emotional factors. It is imperative to look beyond prevalence data to better understand the psychological, socioeconomic, cultural and historical antecedents of self-harm, suicide ideation and suicide to develop more effective suicide prevention strategies.

## 2. Methods and Materials

### 2.1. Search Strategy and Selection Criteria

Eligible studies required: (i) a sample mean age of 24 years or less, (ii) individuals identifying as Australian Aboriginal and/or Torres Strait Islander decent, (iii) a measure of self-harm (i.e., intentional acts of self-injury encompassing both suicide attempts, non-suicidal self-injury or self-injury where intent is unknown or unclear), suicidal ideation (thoughts of engaging in suicide related behaviour and/or planning for suicide) or death by suicide, (iv) publication in English and (v) new data (i.e., not a review of existing data). Unpublished dissertations and Government reports that involved quantitative measures of self-harm, suicidal ideation and/or suicide were included.

This systematic review followed the Preferred Reporting Items for Systematic Reviews and Meta-Analyses (PRISMA) guidelines [23]. Electronic searches of PsychINFO, Web of Science, PubMed and CINAHL databases (from earliest records until April 2019) were conducted using the following search terms, combined with Boolean operators: (“suicid*” OR “suicid* behav*” OR “suicide ideation” OR “at risk” OR “self-harm” OR “self mutilat*” OR “self-injury” OR “substance abuse”) AND (“Aborigin*” OR “Indigenous” OR “Torres Strait Islander”) AND (Australia*) AND (“adolescen*” OR “teen*” OR “youth” OR “young people” OR “child*”).

Initially, three reviewers (K.C., S.D. and C.Mc.) independently screened the titles and abstracts of all identified articles. They then further screened the selected full articles with disagreements arbitrated by two reviewers (J.M.D. and P.J.T.). Reference lists of all articles were checked for additional relevant publications. Authors of the selected review manuscripts were also contacted to see if they had any other possibly eligible papers for inclusion in the review. One additional paper was returned but deemed ineligible according to the criteria. Where identified articles discussed findings concerning suicide and self-harm but specific statistics relating to either Aboriginal or Torres Strait Islander young people were not provided, the corresponding authors of these papers were approached and additional data requested (*n* = 11). We received nine responses and no new relevant information was provided. Data, including study characteristics (e.g., authors, date of publication), study methodology (e.g., design, measures used), participant characteristics and study results (e.g., prevalence or incidence data and associations between variables of interest) were extracted using a spreadsheet template by the researchers to aid synthesis.

### 2.2. Risk of Bias

Included studies were assessed using the Agency for Healthcare Research and Quality methodological quality assessment tool [24]. This tool has been designed to evaluate risk of bias in observational research and was adapted (as in previous studies) for the specific context of this review [25]. The risk of bias assessment for each study is reported in Table 1. We also adopted a narrative synthesis of the risk of bias assessment to report common areas of weaknesses across studies.

## 3. Results

**Figure 1:**
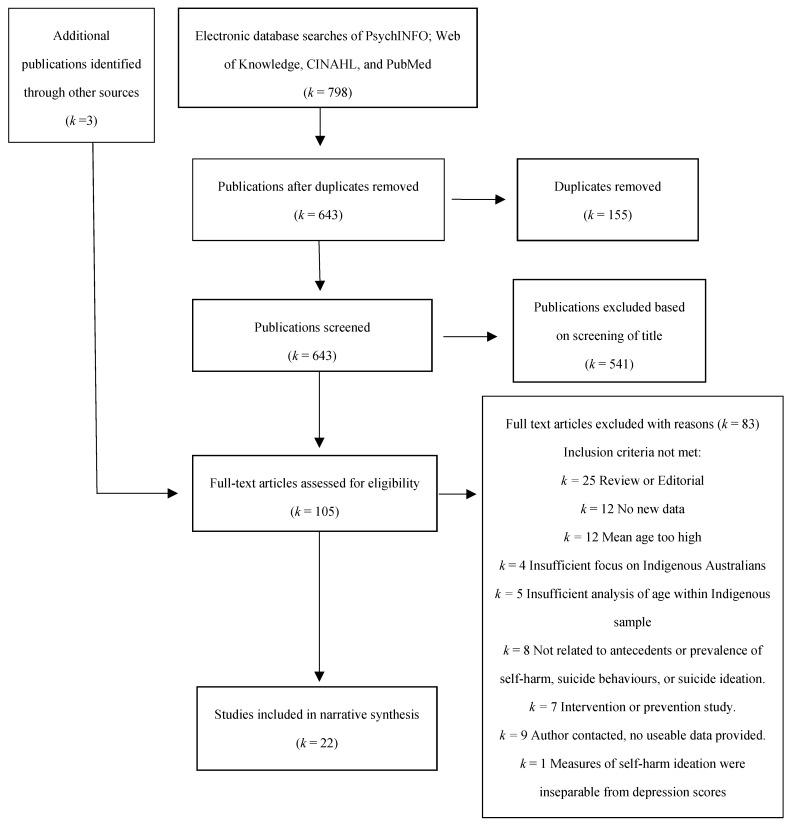
Study selection.

A flow chart of the literature screening is presented in Figure 1. Overall, 22 papers were identified for inclusion in the review. Due to the considerable heterogeneity in study design and measures of suicide, self-harm and suicidal ideation (e.g., self-report, hospital morbidity data and coronial data), aggregation of effect sizes was limited by high heterogeneity and low precision, so meta-analysis was not used here. The results were therefore synthesised narratively. Studies were grouped into five (not mutually exclusive) categories, including studies that evaluated the prevalence of suicide among indigenous youth via analysis of coronial data (*k* = 8), [26,27,28,29,30,31,32,33]; studies that evaluated the prevalence of self-harm and attempted suicide via analysis of hospital admissions records (*k* = 3), [34,35,36]; studies that evaluated the prevalence of suicide, self-harm, or suicide ideation among Indigenous youth in the community (*k* = 7), [37,38,39,40,41,42,43]; studies that involved samples of incarcerated Indigenous youth (*k* = 4), [44,45,46,47]; and studies that evaluated risk factors associated with suicide, self-harm, and suicidal ideation among Indigenous youth (*k* = 5), [33,38,39,40,41]. The characteristics of the studies included in this review are summarised in Table 1.

Sample sizes ranged from *n* = 47 to *n* = 5289. Most studies involved data collected in Western Australia (*k* = 5), [27,34,36,41,45] followed by four studies in the Northern Territory [30,35,38,40], four studies in Queensland, [29,32,33,43] three studies in South Australia [26,28,46] two studies in Victoria [37,39] and one study in New South Wales [44]. Two studies involved data collection from all states and territories in Australia [31,42]. The majority of studies reviewed reported analysis of morbidity or mortality data (i.e., hospital admission records or coronial reports) with a focus on the prevalence of suicide and self-harm (*k* = 11) [26,27,28,29,30,31,32,33,34,35,36]. The majority of studies involved adolescent groups (10–19 years; *k* = 17), [26,27,28,29,32,33,34,35,38,40,41,42,43,44,45,46,47] followed by young adult (15–24 years) (*k* = 11) [27,29,30,31,34,35,36,37,39,42,43] and child samples (0–14 years; *k* = 6). [27,29,32,33,35,43]. The risk of bias assessment for each study is presented in Table 2.

Although several studies did not provide a justification for sample sizes (e.g., power calculations), these studies typically relied on large datasets (10 studies with n > 300) where problems associated with low power are less likely. For the most part, blinding of researchers to participants’ background was not relevant because studies relied on secondary analysis of existing datasets, where rater bias is unlikely to have had an impact (e.g., detection bias associated with an interviewer being more likely to prompt in questions about self-harm if participants have an Aboriginal background). In those studies that relied on interviews or direct assessments to collect study data such biases are possible. However, blinding to ethnicity in studies involving face-to-face interviews may not be realistic. For those studies focused on determining predictors or correlates of suicide, self-harm or suicidal ideation, five studies did not attempt to control for potentially confounding variables. Parameter estimates for these particular studies may be biased as relevant confounders were not taken into account. While most studies used validated measures for determining suicide and self-harm, a common methodological issue associated with the analysis of hospital records was the lack of differentiation between self-harm with suicidal intent and non-suicidal self-injury. For the most part, however, the overall quality of the research conducted was of a good standard.

Eleven studies provided prevalence data estimates for suicide rates among Indigenous relative to nonindigenous youth (see Table 3). Relative to nonindigenous young people, age-standardised prevalence rates were higher for Indigenous populations for both children (Indigenous: 1.5 to 4.61 per 100,000 vs. nonindigenous: 0 to 0.48 per 100,000) and adolescents (Indigenous: 35.6 to 57.50 per 100,000 vs. nonindigenous: 11.7 to 14.33 per 100,000) [29,30]. Studies reported that Indigenous children (15-years of age or less) were between 10 and 14 times more likely to die by suicide, and Indigenous youth (15–24 years) were between four and 14 times more likely to die by suicide than their nonindigenous peers [29,32,33]. Further, the risk of dying by suicide was more than twofold greater for indigenous children than for indigenous adolescents [32].

A recent study identified increasing rates of hospital admissions for intentional self-harm and suicide ideation among Indigenous children living in the Northern Territory, Australia [35]. However, all hospital admissions associated with ICD-10 codes for intentional self-harm and suicidal ideation were analysed as one variable, thus limiting our understanding of the prevalence of self-harm and suicidal ideation, respectively, among this population. These same authors reported that the average annual change in the number of hospital admissions was much greater among Indigenous children (23.5%) than Indigenous youth (11%) and older Indigenous cohorts (25–54 years: 8–13% average annual change).

Seven studies reported prevalence estimates of suicidal ideation among Indigenous youth, with estimates ranging from 9.1 to 46% [32,37,39,40,42,44,46,47].

Time periods over which prevalence rates were estimated however varied between studies (two weeks (*k* = 1), 12 months (*k* = 1), lifetime prevalence (*k* = 2) and not reported (*k* = 3), thus limiting comparisons between studies. One study [37] indicated that almost half (46% *n* = 450) of all calls made to a telephone counselling service by Aboriginal young people related to suicidal ideation or suicide related concerns. Studies also suggested that the prevalence of suicidal ideation was higher among Aboriginal youth who were incarcerated [47] compared to Aboriginal youth living in the community [39,40]. However, amongst incarcerated youth, two studies suggested no difference between Indigenous and nonindigenous detainees or those on remand with regards to suicidal ideation [44,46]. It is possible that ceiling effects account for the lack of differentiation regarding youth suicide ideation in these two studies, as incarcerated youth generally represent a high risk population.

Four studies evaluated the prevalence of self-harm among Indigenous youth [32,36,42,43]. However, cases with and without intent were not differentiated in three of these studies [32,36,43], and suicidal intent was reported in one study [42]. Self-harm was identified as a key issue among Aboriginal youth seeking telephone counselling support, with more than half (59%, *n* = 450) of young Aboriginal callers seeking assistance for self-injury or self-harm related concerns [37]. Age-standardised rates of self-harm, estimated from hospital admissions records suggest that the prevalence of self-harm was much higher among Aboriginal youth (74.23 per 100,000) than for both non-Aboriginal Australian youth (29.18 per 100,000) and young UK born migrants (40.22 per 100,000) [36]. These estimates however were based on data collected between 1984 and 1993. There are also limitations associated with the use of hospital records when estimating the prevalence of self-harm among any population, as a substantial number of self-harm incidents occur that either do not require medical attention or for which medical attention was not sought [11]. Therefore, the rates of self-harm identified by Rock and Hallmayer [36] are likely to be under estimates of self-harm. However, a more recent study comparing three Indigenous communities in Queensland similarly reported a high incidence rate of 30.1 per 1000 for deliberate self-harm presentations among Indigenous young people aged 15–24 years, whereas for those aged 15 years or younger the incidence rate was significantly lower, 1.6 [43]. Lifetime prevalence of actual suicide attempts was found to be significantly higher among Aboriginal or Torres Strait Islander young people aged 18–24 years (14.7; 95% CI 8.0, 25.6) than nonindigenous young people in the same age group (6.3; 95% CI 5.0, 7.9), whereas no significant difference was reported among Indigenous and nonindigenous young people aged between 14 and 17 years (*p* = 0.8) [42]. Risk factors for suicide, self-harm and suicide ideation are presented in Table 4.

Several risk factors for suicide, self-harm and suicide ideation were identified in the systematic review. The findings were all based on cross-sectional designs (see Table 4). Evidence to support sex as a risk factor for suicide and suicidal ideation were mixed. Jamieson and colleagues [38] reported higher levels of suicidal ideation among Indigenous females, relative to Indigenous males, whereas Luke and colleagues [39] reported no significant gender differences. There were however important demographic differences between these studies that may account for the discrepant findings concerning suicide ideation. Luke and colleagues sampled young Indigenous people living in a metropolitan area where a very low proportion of the population identified as Indigenous [39]. In contrast, Jamieson and colleagues (2011) sampled young Indigenous people residing in the Northern Territory where 29.8% of the population identify as Indigenous. Further, another study did not find significant gender differences between young Indigenous males and females in the reported incidence of self-harm [43]; nor was sex associated with increased rates of attempted suicide [39] or death by suicide [32] among Indigenous young people (*k* = 2). However, one study did report higher suicide rates among 5–14 year-old Indigenous males (5–14 years: male= 5.57 per 100,000; female = 3.6 per 100,000) and substantially higher suicide rates among 15–24-year-old males (males = 91.96 per 100,000; females = 22.74 per 100,000), relative to indigenous female [29]. Although Soole and colleagues [32] and De Leo and colleagues [29] extracted data from the Queensland Suicide Register, De Leo and colleagues estimated suicide rates over a much longer time period (1994–2007).

Geographic location was identified as an important risk factor for suicide [33,38]. Particularly high suicide rates were reported among Indigenous children living in remote areas of Australia (Indigenous: 33.75 per 100,000; nonindigenous: 0 per 100,000) [33].

Indigenous children in regional areas were also at a significantly higher risk of suicide than nonindigenous children (Indigenous: 9.5 per 100,000 vs. nonindigenous: 1.4 per 100,000), whereas suicide rates did not differ significantly between Indigenous and nonindigenous children living in metropolitan areas (Indigenous = 0 per 100,000; nonindigenous = 0.56 per 100,000) [33]. Indigenous children were significantly more likely to be residing outside of the parental home prior to suicide and to die by suicide outside of their family home compared to nonindigenous children (*p* = 0.03) [33].

The experience of racial discrimination was identified as a significant risk factor for suicidal ideation among young Aboriginals (β = 0.34) [38,40], particularly among those living in the Northern Territory. One study conducted with Aboriginal youth living in a metropolitan region of Australia (Victoria) identified a trend toward a significant association between lack of cultural connection and suicidal ideation (*p* = 0.06) [39].

Only one study reviewed considered a wide range of psychosocial and cultural factors in relation to risk of suicidal ideation and suicide behaviours [39]. An increased risk of suicidal ideation was associated with emotional distress (OR = 7.6) and social distress (OR = 2.0); aggregate variable encompassed: no friends to talk to, few friends, parents with substance abuse problems, physical abuse and previous youth detention. An increased risk of suicide attempts was also associated with emotional distress (OR = 2.5) and two aggregate social distress variables (OR= 2.5–3.2; encompassing few friendships, no adults to talk to, parents with substance problems, parents not living together, physical abuse, previous youth detention, and low importance of Koori Aboriginal values). Increases in putative risk behaviours (no participation in sport, smoking, heavy drinking, marijuana use) were not associated with increased risk of suicidal ideation; however, there was a trend towards a significant increased risk of suicide attempt (OR = 1.8). Similarly, Jamieson and colleagues [38] reported that risk of suicidal ideation was not related to alcohol or other drug use, but these authors did not investigate risk of suicide attempt. One study investigated the impact of the bereavement, specifically, the death of the birth mother on risk of suicidal ideation and suicide attempts among young Indigenous children [41]. These authors identified an increased risk of suicidal ideation (OR = 2.6) and suicide attempt (OR = 7.0), but not deliberate self-harm (non-suicidal self-injury). This study relied on parent and carer reports, however, which may have underidentified the true rate of self-harm and suicidal ideation.

## 4. Discussion

The current review aimed to synthesise the empirical literature on both the prevalence and antecedents of suicide, self-harm and suicide ideation among Australian Aboriginal and Torres Strait Islander youth. In summary, our main findings were (1) the age standardised suicide rate for Indigenous youth, and Indigenous children in particular, was substantially higher than nonindigenous counterparts; (2) prevalence of self-harm and suicide ideation were higher among Indigenous youth than nonindigenous youth; (3) greater risk of suicidal ideation among Indigenous youth was associated with being incarcerated, experience of racial discrimination, and emotional and social distress, but not substance use; (4) living in regional and remote areas was associated with greater risk of suicide, alongside substance use, being incarcerated and high levels of social and emotional distress were identified as risk factors for suicide; (5) evidence that sex represented a risk factor for suicide and suicidal ideation was inconsistent and there was no evidence available for sex as a risk factor for self-harm; (6) data on prevalence and antecedent risk factors for self-harm among Indigenous youth was limited, although available evidence suggests increased prevalence of self-harm among Indigenous youth compared to nonindigenous youth; and (7) there was a surprising lack of empirical research literature on the antecedent risk factors for suicide, self-harm and suicidal ideation among Indigenous youth.

The current review highlights substantially increased suicide rates among young Aboriginal and Torres Strait Islander youth, relative to nonindigenous Australian youth, thus confirming the emergence of a suicide crisis among young Indigenous populations. We argue the term ‘crisis’ is appropriate, since young Indigenous Australians are not only dying by suicide at significantly higher rates than their nonindigenous peers but they are dying by suicide at an increasingly younger age, particularly in remote regional areas. Suicide rates for young Indigenous Australians aged 15–24 years (39.4 per 100,000) are far higher than the national rate for young people (10.7 per 100,000), [13] and the global suicide rate among young adults 15−29 years, which accounts for 8.5% per 100,000 of all deaths [1]. Prevalence data regarding self-harm in Indigenous Australian youth was more limited. Self-harm is a more difficult phenomenon to monitor, especially at the less medically serious end of the spectrum where individuals may engage in self-harm privately with little contact with social or health services. We argue this crisis extends beyond mental health and encompasses wider social, cultural and emotional factors.

Although much of the available literature has focused on the prevalence of suicide and to a lesser extent suicidal ideation, there is a notable lack of empirical evidence on the antecedents of suicide, self-harm and suicidal ideation among young Australian Indigenous populations. Given that self-harm is a significant predictor of suicide [2,3,4], it is particularly pressing to investigate antecedent risk factors in order to develop more effective prevention and intervention strategies. In particular, it is imperative to identify the key socio-cultural factors (e.g., discrimination and economic deprivation) and psychological factors (e.g., beliefs, thoughts, coping strategies and emotional states) that contribute to elevated risk of suicide and self-harm among Indigenous youth. While this review strongly suggests some candidate variables, including discrimination, social and emotional distress, evaluation of these predictors was limited and no studies examined whether they actually mediated the relationship between belonging to Aboriginal and Torres Strait Islander populations and the risk of suicide, self-harm or suicidal ideation.

Limitations associated with the studies reviewed deserve comment. The lack of longitudinal studies means that the temporal characteristics or direction of the relationship between risk factors and suicide or self-harm is unclear. Western conceptualisations of self-harm may not apply to Aboriginal and Torres Strait Islander populations, as the antecedent thoughts and behaviours that increase the risk of suicide among Indigenous populations may differ from that of the general nonindigenous population [21,48,49,50,51,52]. Those studies that included self-report assessments of self-harm and suicidal ideation, were limited to a brief set of yes/no items (*k* = 3) or a single item assessment (*k* = 1). Further, assessment of suicidal ideation and/or self-harm was not adequately described (*k* = 3), e.g., the works by the authors of [39,40,46]. As such, a more detailed examination of the frequency and severity of these phenomena is precluded. Future research would benefit from the development of more culturally sensitive and appropriate measures to assess suicide risk.

The over representation of studies reporting morbidity and mortality data in the current review reflects the accessibility of this population level data. Indigenous suicides are often not effectively identified by coroners and therefore there may be significant under reporting [20]. Further, there is a surprising lack of qualitative research. The current review did not exclude qualitative methods from the search strategy, however, no qualitative research designs or assessments of suicide, self-harm or suicidal ideation among Aboriginal and Torres Strait Islander youth were identified. It could be argued that it may be more effective to undertake qualitative research in a culturally sensitive manner (e.g., narrative analysis) as these approaches seek to understand the ‘insider’ experience and complexity of a particular phenomenon. Meta-analysis would have helped to derive more precise estimates of prevalence and associations with risk factors, but was not possible due to substantial heterogeneity in study designs and outcomes and the likely nonindependence of studies relying on large national datasets.

Based on the present systematic review, the findings highlight some key avenues for future research and public health policy. Improvement in routine collection of self-harm information via hospital and mental health service providers, along with standardised reporting systems would allow for national level statistics to improve prevalence estimates. Large scale longitudinal studies would provide a better test of predictors of risk, for both suicidal ideation and self-harm among Aboriginal and Torres Strait Islanders. Health and social practitioners working with Aboriginal and Torres Strait Islander communities should be mindful of the elevated suicide and self-harm risk factors. It has been shown that psychosocial interventions can help reduce self-harm risk [53], but trials of culturally adapted interventions that specifically target self-harm in Aboriginal and Torres Strait Islander populations are sparse [54]. A trial of a self-help mobile phone application, designed with Aboriginal and Torres Strait Islander people, aimed at reducing suicide ideation is currently ongoing [55]. Given the high proportion of Indigenous people living in regional and remote areas, and the high incidence of youth suicide in such areas, a research agenda aimed at profiling the unique psychological, social and cultural needs of these communities in relation to suicide and self-harm is required. Research aimed at studying self-harm, suicide ideation and suicide among incarcerated youth awaits further investigation. Many Aboriginal and Torres Strait Islander communities are attempting to tackle the serious crisis of youth suicide [51,52]. Aboriginal communities are best positioned to identify their specific research questions to better understand the antecedents of youth self-harm, suicide ideation and suicide, and the development of more effective preventive strategies and public policies, within their specific communities. Given many of the risk factors identified in this review are social or societal in nature, including discrimination or environment, broader social policy initiatives may also be an important step in reducing self-harm and suicide. These may include initiatives to reduce discrimination, increase social cohesion, preserve culture and promote quality of life and self-determination in the community.

## 5. Conclusions

This review highlights the substantially elevated rates of suicide, self-harm and suicidal ideation amongst Aboriginal and Torres Strait Islander young people in Australia compared to nonindigenous young people. These elevated rates reflect the complex range of social, cultural and psychological adversities faced by the Aboriginal and Torres Strait Islander youth population in Australia, but further research is needed to delineate these factors. The findings strongly support and justify the increasing call for the implementation of public health programs specifically aimed at tackling the crisis of suicide and self-harm among Aboriginal and Torres Strait Islander young people [51,52,56,57,58]. Such programs will be strengthened by a greater recognition of the specific social, cultural, psychological and intergenerational determinants of self-harm and suicide among Aboriginal and Torres Strait Islander young people. Aboriginal and Torres Strait Islander communities need to be at the forefront in shaping and implementing these programs to tackle this urgent crisis.

## Figures and Tables

**Table 1:** Quality assessment.

Title	Unbiased Sample Selection	Selection Minimise Baseline Difference	Sample Size Calculated	Adequate Description of Cohort	Aboriginal Status Clearly Stated	Validated Method for Ascertaining Suicide/Self-Harm	Blind to Exposure	Adequate Follow up Period	Minimal Missing Data	Analysis Controls for Confounding	Analytic Methods Appropriate
Adams et al. (2014) [37]	Yes	n/a	n/a	Partial	Yes	Partial	n/a	n/a	Yes	n/a	n/a
Armstrong et al. (2017) [42]	Yes	n/a	n/a	Yes	Yes	Yes	n/a	n/a	Yes	n/a	Yes
Burvill (1975) [34]	Yes	n/a	n/a	Yes	Yes	Yes	n/a	n/a	Yes	n/a	Yes
Byard et al. (2011) [26]	Yes	n/a	n/a	Yes	Yes	Yes	n/a	Partial	Yes	n/a	n/a
Campbell et al. (2016) [27]	Yes	n/a	n/a	Yes	Yes	Yes	n/a	n/a	Yes	n/a	Yes
Clayer & Czechowicz (1991) [28]	Yes	n/a	n/a	Yes	Yes	Yes	n/a	n/a	Yes	n/a	n/a
De Leo et al. (2011) [29]	Yes	n/a	No	Yes	Yes	Yes	n/a	Partial	Yes	n/a	Yes
Fascher et al. (1997) [44]	Yes	n/a	n/a	Yes	Yes	Partial	No	n/a	Yes	n/a	n/a
Jamieson et al. (2011) [38]	Yes	n/a	No	Yes	Yes	Yes	n/a	Partial	Yes	Yes	Yes
Lawlor & Kosky (1992) [45]	Yes	n/a)	n/a	Yes	Yes	Yes	n/a	n/a	Yes	n/a	n/a
Leckning et al. (2016) [35]	Yes	n/a	No	Yes	Yes	Yes	n/a	n/a	Yes	n/a	Yes
Luke et al. (2013) [39]	Yes	n/a	No	Yes	Yes	Partial	No	n/a	Yes	Yes	
Pridmore & Fujiyama (2009) [30]	Yes	n/a	No	No	Yes	Yes	n/a	n/a	Yes	n/a	Yes
Priest et al. (2011) [40]	Yes	n/a	Partial	Yes	Yes	Partial	No	n/a	No	Yes	Yes
Robinson et al. (2016) [31]	Yes	n/a	No	No	Yes	Yes	n/a	Yes	Yes	n/a	Yes
Rock & Hallmayer (2008) [36]	Yes	n/a	No	Partial	Yes	Yes	n/a	Yes	Yes	No	Yes
Rouen et al. (2019) [43]	Yes	n/a	n/a	Partial	Yes	Yes	n/a	n/a	Yes	n/a	Yes
Sawyer et al. (2010) [46]	Yes	No	No	Yes	Yes	Partial	No	n/a	Yes	No	Yes
Soole et al. (2014a) [32]	Yes	n/a	No	Yes	Yes	Yes	n/a	n/a	Yes	n/a	Yes
Soole et al. (2014b) [33]	Yes	n/a	No	Yes	Yes	Yes	n/a	n/a	Yes	No	Yes
Stathis et al. (2012) [47]	Yes	n/a	No	Partial	Yes	Yes	No	n/a	Yes	No	Yes
Zubrick et al. (2011) [41]	Yes	n/a	No	Yes	Yes	No	No	n/a	unclear	No	Yes

**Table 2:** Study Characteristics.

Author	Sample	Data Period	State of Australia	Method	Measure or Suicidality/Self-Harm
Adams et al. (2014) [37]	Total calls to Kids Helpline: *N* = 16,687 (5–25 years) Aboriginal: *N* = 450 calls Non-Aboriginal: *N* = 16,237 calls	2012	Victoria (VIC)	Analysed data from Kids Helpline, a counselling service for young people	Call categorisation by call centre workers
Armstrong et al. (2017) [42]	Indigenous males: *N* = 432 (2.7% of sample) Nonindigenous males: *N* = 15,859 (97.3% of sample) 14–17 years *n* = 48 Indigenous males *n* = 1135 Nonindigenous males18–24 years *n* = 71 Indigenous males *n* = 1853 Nonindigenous males	2013–2014	All	Relevant cross-sectional prevalence data extracted from large-scale cohort study of Australian males aged a 10-years and older. The Australian Longitudinal Study on Male Health (Ten to Men)	Item 9 (PHQ-9) to assess prevalence of suicidal thoughts. Single item to assess suicide attempts
Burvill (1975) [34]	Total attempted suicides: *N* = 2036; (age range: 15–24) Aboriginal: *N* = 18 Non-Aboriginal: *N* = 2018	1971–1972	Western Australia (WA)	Examined hospital data on attempted suicide rates in Perth	Hospital morbidity data
Byard et al. (2011) [26]	Total adolescent deaths: *N* = 522 Suicide attributed to asphyxia: *N* = 62; Mage 16.6 years (SD not reported) (age range: 10–18) Breakdown by ethnicity not reported.	1994–2010	South Australia (SA)	Examined Coronial reports of adolescent asphyxial deaths	Coroner’s reports
Campbell et al. (2017) [27]	Reported deaths by suicide: *N* = 125 (age range: 0–14; 15–19; 20–24) Indigenous suicide: *N* = 102 Nonindigenous suicide: *N* = 16 Ethnicity not known: *N* = 7	2005–2014	Western Australia (WA)	Audit of suicide data provided to Kimberly Mental Health and Drug Service	Coroner’s reports
Clayer & Czechowicz (1991) [28]	Total Suicides: *N* = 125 (age range: 10–19) Aboriginal: *N* = 15 (12 Male) Non-Aboriginal: *N* = 110 (93 Male)	1981–1988	South Australia (SA)	Examined coroner’s records	Coroner’s reports
De Leo et al. (2011) [29]	Not Reported	1994–2007	Queensland (QLD)	Analysis of Queensland Suicide Register	Coroner’s reports
Fasher et al. (1997) [44]	Total detainees: *N* = 100; Mage: 15.9 ± 1.37 (range 12–18) Aboriginal *N* = 30 Non-Aboriginal: *N* = 70	1994	New South Wales (NSW)	Analysed nursing admission forms and semistructured interviews with detainees at Cobham Juvenile Justice Centre	Nursing admission assessment forms. Suicide ideation recorded as positive for any individual who reported ever having thought about suicide. Attempted suicide recorded as positive for any individual who reported that they had attempted suicide. Self-injurious behaviour referred to physical injury caused by impulsive behaviour while angry
Jamieson et al. (2011) [38]	Aboriginal young people: *N* = 336; age range <18 years.	1987–1990	Northern Territory (NT)	Longitudinal analysis of mental & dental health of birth cohort from Royal Darwin Hospital	Strong Souls questionnaire. Suicide risk rated on 10-point scale, 3 items (have you wished you were dead? Have you felt like hurting yourself? Have you felt like killing yourself? Prevalence = past few months’
Lawlor & Kosky (1992) [45]	Total Suicide attempts: *N* = 12 Non-suicidal adolescents: *N* = 30 (age range= 11.11–17.10 years; Mage: 16.9, SD not reported) Aboriginal suicide attempts: *N* = 4 Non-Aboriginal suicide attempts: *N* = 8	1987–1989	Western Australia (WA)	Analysed records of attempted suicide at Longmore Remand Centre	Longmore Remand Centre records of attempted suicide. Suicidal attempt defined as a suicide attempt which could have led to death if intervention had not been successful. Information on past suicide attempt and suicide ideation obtained from staff reports of “unusual occurrences”
Leckning et al. (2016) [35]	Hospital admissions for intentional self-harm and suicidal ideation *N* = 4495. (age-range: 10–24) Indigenous: *N* = 2367 admissions. Nonindigenous: 2127 admissions not reported: *N* = 2. No information reported for number of young person hospital admission	2001–2013	Northern Territory (NT)	Examination of hospital dataset for admissions related to intentional self-harm or ideation via ICD-10 codes	Analysis of hospital records. Intentional self-harm (ICD-10-AM codes X60-X84) and suicidal ideation (ICD-10-AM code R45.81)
Luke et al. (2013) [39]	Aboriginal (Koori) young people: *N* = 172; (age range 12–26; *M* age: 19 ± 4); (75 Male) No Non-Aboriginal group.	1997–1998	Victoria (VIC)	VAHS Young People’s Project. Data from health questionnaire and health check	Adapted Gatehouse Survey questionnaire. Suicide ideation: In the last two weeks, have you had thoughts that you would be better off dead in some way?; Suicide attempt: Have you ever tried to kill yourself?; (Y/N)
Pridmore & Fujiyama (2009) [30]	Total suicides reported in NT: *N* = 256 Age range: (<15–24) Indigenous: *N* = 130 Nonindigenous: *N* = 126	2001–2006	Northern Territory (NT)	Examined National Coroner’s Information System	Coroner’s reports
Priest et al. (2011) [40]	Aboriginal young people: *N* = 345; (age range 16–20.5; Mage: 18.27 ± 1.06); (162 Males)	2006–2008	Northern Territory (NT)	Cross-sectional analysis of Aboriginal Birth Cohort Study	Strong Souls questionnaire. Suicide risk assessed via 3 items (details not reported). Binary coding (0 = present; 0 = not present)
Robinson et al. (2016) [31]	Youth suicides: *N* = 940 (age: <24 years)	2010–2012	National	Analysis of deaths classified as intentional self-harm; National Coronial Information System	National Coronial Information System
Rock & Hallmayer (2008) [36]	Suicide: 15–24 years Indigenous: *N* = 24 (23 males, 1 female) Nonindigenous: *N* = 305 (262 males, 43 females) Self-harm: 15–24 years Indigenous self-harm: *N* = 643 (265 males, 378 females) Nonindigenous: *N* = 5400; (2141 males, 3259 females)	1984–1993	Western Australia (WA)	Analysed data extract from Western Australian linked case registers of suicide and deliberate self-harm in Aboriginals, Australians & UK Migrants	Linked case registers: hospitalisations with an ICD-9-CM diagnosis of deliberate self-harm (E950–958). Suicide (E950–958) on Register of Births, Deaths, and Marriages
Rouen et al. (2019) [43]	Indigenous sample from 3 communities (*N* = 2262) Community A (*n* = 1063) Community B (*n* = 578) Community C (*n* = 621)	2006–2011	N. Queensland	Audit over 10–14 days re self-harm data from clinical files in community primary health care centres by registered nurse	ICD-10-CM: 2014. Classification of Diseases—Injury Classification System
Sawyer et al. (2010) [46]	Adolescents remanded into custody: *N* = 159 (age-range: 13–17 years) Aboriginal: *N* = 52 Non-Aboriginal: *N*= 107	2008–2009	South Australia (SA)	Cross-sectional analysis of adolescents remanded to Magill Youth Centre	Youth Risk Behaviour Surveillance System Questionnaire. Experience of suicidal ideation, plan for suicide, attempted suicide, and/or initiated a suicide attempt which required medical treatment (Y/N)
Soole et al. (2014a) [32]	Child/adolescent deaths due to external causes: *N* = 469 (age range: 10–17 years); (308 male) Indigenous: *N* = 42; (age range: 10–17) Nonindigenous: *N* = 99; (age range: 10–17 years)	2004–2012	Queensland (QLD)	Analysis of Queensland Suicide Register (QSR)	Causes of death classified according to ICD-10 (self-harm X60-X84)
Soole et al. (2014b) [33]	Child deaths by suicide: *N* = 45; (age range: 10–14 years); (25 Male) Indigenous: *N* = 21; *M*age: 13.05 (SD not reported); (12 Male)	2000–2010	Queensland (QLD)	Analysis of Queensland Suicide Register (QSR)	QSR data cross-referenced with National Coronial Information System
Stathis et al. (2012) [47]	Indigenous young people in custody: *N* = 47; (Mage 14.5 ± 1.3 years); (37 Male) No nonindigenous group	Not reported	Not specified	Cross-sectional assessment of admissions to MHATODS	Westerman Aboriginal Symptoms Checklist—Youth

International Classification of Disease (ICD-9; ICD-10); Mental Health Alcohol Tobacco and Other Drugs Service (MHATODS); Victorian Aboriginal Health Service (VAHS).

**Table 3:** Prevalence of suicide, self-harm and suicide ideation.

Author	Suicide	Self-Harm	Suicide Ideation
Adams et al. (2014) [37]	Not Investigated	Fifty-nine percent of Aboriginal callers sought assistance for self–injury and/or self-harm concern.	Forty-six percent of Aboriginal callers reported suicide related concern and/or ideation
Armstrong et al. (2017) [42]	Not Investigated	18–24 years: significantly higher prevalence rate of suicide attempts among Indigenous males (14.7%; *n* = 11/71) than non-Indigenous males (*n* = 116/1853; 6.3%) OR = 2.5, 95% CI = 1.2, 5.2 (*p* = 0.014) 14–17 years: prevalence of suicide attempts among Indigenous (3.4%) (*n* = 2/48) and non-Indigenous males (3.6%) (*n* = 43/1135) OR = 0.9, 95% CI = 0.2, 4.4 (*p* = 0.862), non–significant.	18–24 years: no significant difference in prevalence of suicidal thoughts among Indigenous males (10.8%; *n* = 7/70) than non-Indigenous males (11.4%; *n* = 214/1850); OR = 0.9, 95% CI = 0.4, 2.4 (*p* = 0.877). 14–17 years: no significant difference in prevalence of suicidal thoughts among Indigenous males (9.1%; *n* = 11/98) and non-Indigenous (9.0%; *n* = 163/1943); OR = 1.0, 95% CI = 0.4, 2.3 (*p* = 0.994).
Burvill (1975) [34]		15–24 years: a rate of 21 per 10,000 attempted suicides per male Aboriginals and a rate of 74 per 10,000 attempted suicides per female Aboriginals between 1971 and 1972 in Perth, West Australia.	Not Investigated
Byard et al. (2011) [26]	Asphyxia reported as cause of death for 19.4% (*n* = 12) of Aboriginal suicides and 80.6% (*n* = 50) of suicides for non-Indigenous people.	Not Investigated	Not Investigated
Campbell et al. (2016) [27]	48% *(n* = 71) of Indigenous deaths by suicide were reported among those aged <20 years.	Not Investigated	Not Investigated
Clayer & Czechowicz (1991) [28]	32.6% (*n* = 15) of Aboriginal suicides occurred among 10–19 year olds compared to 8.4% (*n* = 110) of non-Aboriginal young people of the same age range.	Not Investigated	Not Investigated
De Leo et al. (2011) [29]	5–14 year olds: Indigenous: 4.61 per 100,000 vs. non-Indigenous 0.48 per 100,000 (Risk Ratio 9.6, 95%CI = 1.24–74.3) 15–24 year olds: Indigenous: 57.50 per 100,000 vs. non-Indigenous 14.33 per 100,000 (Risk Ratio 4.0, 95% CI = 2.24–7.18).	Not Investigated	Not Investigated
Fasher et al. (1997) [44]		No significant difference in the proportion of Aboriginal (6.7%) and non-Aboriginal detainees (10.0%) reporting a history of attempted suicide (*p* = 0.594)	No significant difference in the proportion of Aboriginal (23.3%) and non-Aboriginal (27.1%) detainees reporting suicidal ideation (*p =* 0.691)
Lawlor & Kosky (1992) [45]		Majority of suicide attempts were made by Non-Indigenous adolescents. 33.3% (*n* = 4/12) of suicide attempts were made by Aboriginal adolescents.	Not Investigated
Leckning et al. (2016) [35]		Average percentage increase (2001–2013) in rates of hospital admission for suicidal behaviour in children aged 10–14 years was greater for Indigenous populations (23.5%) than non-Indigenous (12.8%) Average percentage increase (2001–2013) in rates of hospital admission for suicidal behaviour in young people aged 15–24 years was greater for Indigenous populations (11.0%) than non-Indigenous (6.6%) (*p* < 0.05).	
Luke et al. (2013) [39]		24.4% *(n* = 42) reports of past suicide attempt. Sig greater number of suicide attempts among 17–21 (31.6%) and 22–26 (30%) age range than <16 age range (4.7%) (*p* = 0.002)	23.2% (*n* = 40) reported suicidal ideation in previous two weeks. Sig higher prevalence of suicidal ideation reported by Aboriginal youth aged 17–21 years than to <16 years (*p* = 0.008)
Pridmore & Fujiyama (2009) [30]	Suicide rates for Aboriginal children (<15 years) was 1.5 per 100,000 compared to 0 per 100,000 for non-Indigenous. Young people (15–24 year olds) was 35.6 per 100,000 compared to 11.7 per 100,000 for non-Indigenous youth.	Not Investigated	Not Investigated
Priest et al. (2011) [40]	25% (*n* = 87) of young Aboriginal people surveyed reported a risk of suicide.	Not Investigated	
Robinson et al. (2016) [31]	Suicides by Indigenous young people were significantly more likely to occur in a cluster (58.5%) than suicides by non-Indigenous people (13.1%) (*p* < 0.001)	Not Investigated	Not Investigated
Rock & Hallmayer (2008) [36]	Suicide rate for the 15–24 year olds Aboriginal population was 2.77 per 10,000 compared to 1.65 per 10,000 for non-Aboriginal Australians, and 2.31 per 10,000 for UK born migrants.	Self-harm rates were 74.23 per 10,000 for 15–24 year old Aboriginal population, compared to 29.18 per 10,000 for non-Aboriginal Australians, and 40.22 per 10,000 for UK born migrants.	Not Investigated
Rouen et al. (2019) [43]	Not Investigated	15–24 years: significantly higher incidence rate of self-harm presentations (per 1000 population) among Indigenous young people in this age group, IR 30.1 (*n* = 71 presentations), CI = 23.1–37.1, relative to Indigenous young people aged 15 years and younger, IR 1.6 (*n* = 6 presentations) CI = 0.3–2.9 (*p* < 0.001). Rates of self-harm not significantly different between genders.	Not Investigated
Sawyer et al. (2010) [46]	No sig difference in prevalence of suicide behaviour between Aboriginal and non-Aboriginal adolescents on remand (*p*-values not reported).	Not Investigated	No significant difference in prevalence of suicidal ideation between Aboriginal and non-Aboriginal adolescents on remand (*p*-values not reported).
Soole et al. (2014a) [32]	Indigenous children and adolescents were sig more likely to die by suicide than by other external causes (OR = 14.47, 95% CI = 4.63–45.20 for children; OR = 2.89, 95% CI = 1.54–5.40 for adolescents) Indigenous children sig more likely to die by suicide aged 10–14 years than aged 15–17 years (OR = 2.68, 95% CI = 1.18–6.08). No sex difference in rate of suicide 0–14 year olds (male OR = 0.89; female OR = 1; 95% CI 0.27–2.88.	No sig difference in the percentage of indigenous children (25.8%, *n* = 8) and indigenous adolescents (30.5%, *n* = 32) reporting previous self-harm (OR = 0.79, 95% CI = 0.32–1.96)	No sig difference in percentage of indigenous children (29.4, *n* = 10) and indigenous adolescents (41.7%, *n* = 45) reporting previous suicidal ideation (OR = 58, 95% CI = 0.25–1.34) indigenous adolescents.
Soole et al. (2014b) [33]	Suicide rate for Indigenous children 10.63 per 100,000 (11.23 for males and 9.00 for females) compared to <1 per 100,000 (0.85 for males and 0.76 for females) (RR = 12.63, 95% CI = 6.77–23.51).	Not Investigated	Not Investigated
Stathis et al. (2012) [47]	Not Investigated	Not Investigated	34% (*n* = 18) of Indigenous youth reported suicidal ideation. Indigenous females were sig more likely to report suicidal thoughts than males (*p* < 0.001; *d* = 0.78).

Note: 95% confidence interval (95% CI); Odds Ratio (OR); Risk Ratio (RR); Incidence Rate (IR). Data analysis combined IDC-10 codes for self-harm with or without intent and suicide ideation. *d* = effect size.

**Table 4:** Risk factors for suicide, self-harm and suicide ideation.

Author	Suicide	Self-Harm	Suicide Ideation
Jamieson et al. (2011) [38]	Not Investigated	Not Investigated	Young Aboriginal females reported sig higher levels of suicidal ideation than young Aboriginal males (β = 0.49, 95% CI = 0.25–0.74). Aboriginal youth experienced racial discrimination were at a significantly higher risk of suicide ideation than those who did not (β = 0.34, 95% CI = 0.08–0.60). No significant difference in level of suicidal ideation among Aboriginal youth living in regional and remote areas. No significant difference in level of suicide ideation among Aboriginal youth who were employed vs. unemployed. No significant difference in level of suicidal ideation among Aboriginal youth who use tobacco, marijuana, or alcohol and those who do not.
Luke et al. (2013) [39]	No significant difference between males and females in number of past suicide attempts (*p* = 0.123). Suicide attempt 2.5 times greater (95% CI = 0.45–4.46, *p* = 0.001) with an increase of 1 unit in emotional distress. Suicide attempt 3.2 times greater (95% CI = 1.75–6.05; *p* < 0.001) with an increase of one unit in social distress B (see footnote). Suicide attempt 2.5 times greater (95% CI = 1.38–4.62, *p* = 0.003) with an increase of one unit in social distress A (see footnote). No significant association between cultural connection and past suicide attempt (*p* = 0.370). Trend towards significant association between behavioural component and past suicide attempt (OR = 1.82, 95% CI = 0.99–3.37, *p* = 0.055).	Not Investigated	No significant difference between males and females in level of suicidal ideation (*p* = 0.872). Suicide ideation 7.6 times greater (95% CI = 3.41–16.95, *p* < 0.001) with an increase of 1 unit in emotional distress. Suicide ideation 2.0 times greater (95% CI = 1.15–3.65, *p* = 0.015) with an increase of one unit in social distress B. No significant association between cultural connection and suicidal ideation (*p =* 0.062). No significant associations between behavioural component and level of suicidal ideation (OR = 1.47, 95% CI = 0.78–2.78, *p* = 0.22)
Priest et al. (2011) [40]	Not Investigated	Not Investigated	Racial discrimination associated with significant increased risk of suicidal ideation (OR, 2.32 (95% CI, 1.25–4.00; *p* = 0.001)).
Soole et al. (2014b) [33]	Rates of suicide among indigenous children higher in remote (33.75 per 100,000) than regional (9.54 per 100,000) and metropolitan areas (0 per 100,000). Suicide rates higher among indigenous than non-Indigenous in remote and regional areas (non-Indigenous: remote = 0 per 100,000; regional = 1.4 per 100,000; RR = 6.81, 95% CI = 2.76–16.50, *p* < 0.001). In metropolitan areas, low rates among indigenous and non-Indigenous children (0.56 per 10,000). Significantly higher number of Indigenous children consumed alcohol prior to suicide compared to non-Indigenous children (33.3%, *n* = 7, 4.2%, *n* = 1, *p* = 0.014). No significant difference in physical health conditions between indigenous and non-Indigenous children who died by suicide (*p* = 0.267).	Not Investigated	Not Investigated
Zubrick et al. (2011) [41]	Aboriginal children whose birth mother had died were at a sig higher risk for parent-reported suicide attempts (OR = 7.0, 95% CI = 1.6–31.1 *p* value not reported).	No significant difference between Aboriginal children who lived with their birth mother or whose birth mother had died with regards to self-harm.	Aboriginal children whose birth mother had died were at a sig higher risk for parent-reported suicidal ideation (OR = 2.6, 95% CI = 1.2–5.7; *p* value not reported).

Note: 95% confidence interval (95% CI); Odds Ratio (OR); Risk Ratio (RR). Outcome of Luke et al. (2013) principle component analysis identified five uncorrelated components. These were categorised as emotional distress (component 1) (depression, anger, boredom, poor self-esteem and sexual abuse); social distress A (component 2) (Koori Aboriginal values not important, parents not living together, no adults to talk to, few friends, parents with substance problems, physical abuse and previously in youth detention); social distress B (component 3) (no friends to talk to, few friends, parents with substance problems, physical abuse, previously in youth detention); cultural connection (component 4) (talk to elders about Koori Aboriginal issues; understand Koori history, use Victorian Aboriginal Health Service as main service provider and parents have high expectations); and behavioural (component 5) (no participation in sport, smoker, heavy drinker and marijuana use).
